# ﻿*Camelliashuangbaiensis* (Theaceae), a new species from Yunnan, China

**DOI:** 10.3897/phytokeys.254.144891

**Published:** 2025-03-26

**Authors:** Zi-Yuan Li, Bao-Huan Wu, Shang Qu, Le-Shan Du, Hai-Ou Liu, He-Xiang Duan, Fei-Fei Li, Wen-Hui Liu

**Affiliations:** 1 Institute of Ecology, Chinese Research Academy of Environmental Sciences, Beijing 100012, China Institute of Ecology, Chinese Research Academy of Environmental Sciences Beijing China; 2 Guangzhou Institute of Forestry and Landscape Architecture, Guangzhou 510405, China Guangzhou Institute of Forestry and Landscape Architecture Guangzhou China; 3 Guangzhou Horticultural Plant Germplasm Resource Nursery, Guangzhou 510405, China Guangzhou Horticultural Plant Germplasm Resource Nursery Guangzhou China; 4 Guangzhou Collaborative Innovation Center on Science-tech of Ecology and Landscape, Guangzhou 510405, China Guangzhou Collaborative Innovation Center on Science-tech of Ecology and Landscape Guangzhou China; 5 Beijing Adonis Environmental Protection Technology Co., LTD, Beijing 100035, China Beijing Adonis Environmental Protection Technology Co., LTD Beijing China; 6 Yunnan Research Academy of Eco-environmental Sciences, Kunming 650034, China Yunnan Research Academy of Eco-environmental Sciences Kunming China; 7 Beijing Botanical Garden, Beijing, 100093, China Beijing Botanical Garden Beijing China; 8 Key Laboratory of National Forestry and Grassland Administration on Plant Ex situ Conservation, Beijing, 100093, China Key Laboratory of National Forestry and Grassland Administration on Plant Ex situ Conservation Beijing China

**Keywords:** *Camellia*, Flora, new taxon, Shuangbai County, taxonomy

## Abstract

A new species of the genus *Camellia* (Theaceae), *Camelliashuangbaiensis* G.P.Yang & B.H.Wu, **sp. nov.**, from the central region of Yunnan Province of China is described. *Camelliashuangbaiensis* is morphologically similar to *C.mileensis* and *C.hongkongensis*, but it can be distinguished by its smaller leaves with an ovate, abaxially tomentose lamina, and 14–16 bracteoles and sepals.

## ﻿Introduction

*Camellia*Linnaeus (1753: 698) (Theaceae) is a genus of evergreen trees or shrubs predominantly distributed across the tropical and subtropical regions of Asia (Chang and Ren 1998; Ming and Bartholomew 2007). Renowned for their ornamental value and economic importance, particularly in tea production, *Camellia* species have been cultivated for a long history in China, and China harbors the richest diversity of *Camellia*, with over 80% of the recorded species within its region (Chang and Ren 1998; Ming and Bartholomew 2007). From 2020 to 2025, there were 8 new *Camellia* species described from China (Liu et al. 2020a, 2020b; Xu et al. 2020; Yu et al. 2021; Ye et al. 2022; Zhang et al. 2022; Chen et al. 2023; Lin et al. 2024). Moreover, a study using floral pigments and multivariate analyses suggested that the Xinan District in China, encompassing Yunnan province, is presumed to be the origin site of red-flowered *Camellia* species (Li et al. 2013).

During our field investigations in Yunnan Province in 2024, we found an unknown *Camellia* species with red flowers with 3 distinct styles and brown fruits with furfuraceous surfaces. Through extensive morphological comparisons and taxonomic analyses, we have confirmed that these specimens represent a new species, which we formally describe in this document.

## ﻿Materials and methods

Morphological comparisons of the putative new species with related species were conducted using living plants, relevant literature, and herbarium specimens. Measurements were conducted manually with rulers or using Digimizer version 4.6.0 (MedCalc Software, Mariakerke, Belgium). The voucher specimens were deposited in the herbarium of China National Botanical Garden (**CNBG**), the herbarium of South China Botanical Garden (**IBSC**) and the herbarium of Sun Yat-sen University (**SYS**).

## ﻿Results

### ﻿Taxonomic treatment

#### ﻿ Camellia shuangbaiensis

Taxon classificationPlantaeEricalesTheaceae

G.P.Yang & B.H.Wu sp. nov.

9C3538D1-E281-5ACB-8FA8-EFDF9F8F78C4

urn:lsid:ipni.org:names:77359329-1

**Figure 1.**
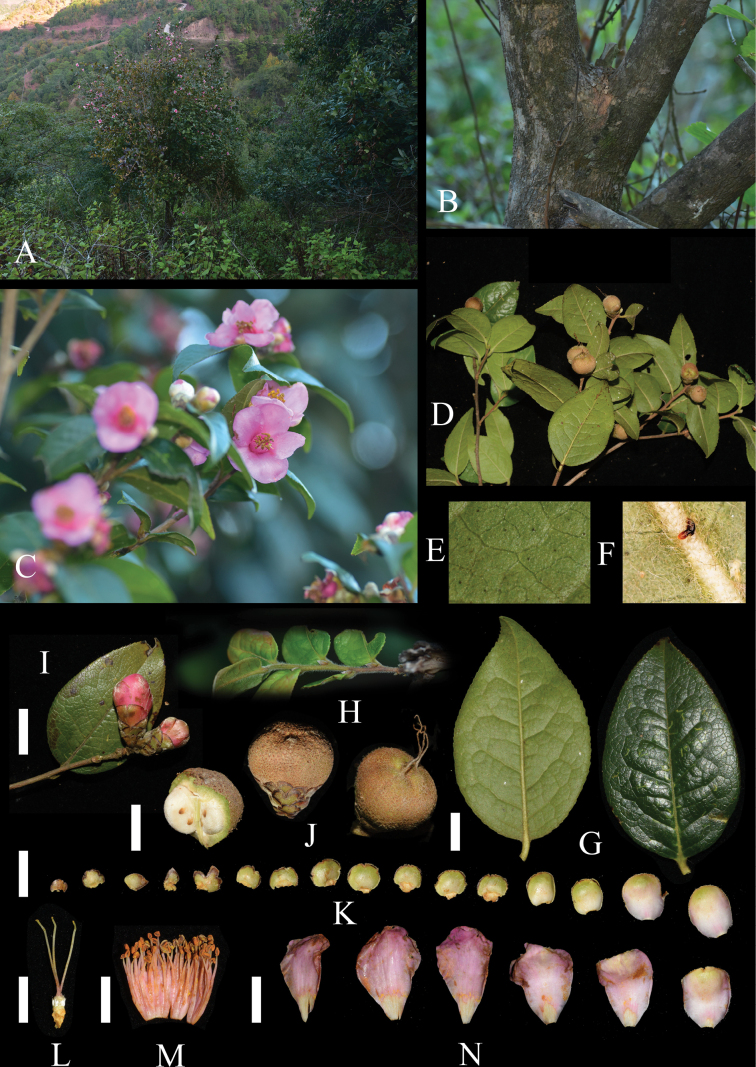
*Camelliashuangbaiensis***A** flowering individual and habitat **B** stem **C** flowering branch **D** fruiting branches **E** tomentose and punctate leaf abaxial surface **F** pubescent midvein on the leaf abaxial surface **G** leaves **H** branchlet **I** flower buds **J** fruits **K** bracteoles and sepals **L** pistil **M** androecium **N** petals. Photographed by Shang Qu and Bao-Huan Wu. Scale bars: 1 cm.

Fig. 1 Chinese name: 双柏山茶 (Shuang Bai Shan Cha)

##### Type.

**China** • **Yunnan**: Shuangbai County, Damaidi Township, in ravine. 24°22.44'N, 101°50.84'E, 1740.262051 m a.s.l., 6 June 2024 (fl.), *S. Qu and G.P. Yang Lg2024132* (holotype: CNGB!; isotypes: IBSC!).

##### Diagnosis.

*Camelliashuangbaiensis* morphologically resembles *C.mileensis* T.L.Ming and *C.hongkongensis* Seem., but it can be distinguished from the latter two species by its leaves with ovate shape, tomentose abaxial surface and rounded leaf base and bracteoles/sepals 14–16.

##### Description.

Small evergreen tree, 2–4 m tall; bark greyish brown, rough; current-year branchlets densely covered with whitish pubescence. Leaf blades ovate to elliptic, leaf apex acuminate to acute, leaf base round, 3.5–6.5 × 2.3–4 cm, leaf blade leathery, adaxially dark green, pubescent along the midrib, abaxially light green, tomentose, more or less punctate; midrib prominent on both surfaces, secondary veins 5–6 pairs, elevated on adaxial surface and impressed on abaxial surface; petiole 2–5 mm long, pubescent. Flowers solitary or sometimes 2 or 3-clustered, terminal, subterminal or axillary, 2–4 cm in diameter, sessile. Bracteoles and sepals 14–16, semipersistent, outside brownish silky pubescent, inside glabrous; outer bracteoles and sepals broadly semiorbicular, rarely apex bifid; inner bracteoles and sepals suborbicular to oblong-elliptic. Petals 6–7, red, basally slightly connate, oblong-elliptic to obovate-elliptic, apex round, 7–11 × 4–5 mm, outside white silky pubescent along ridge. Stamens 33–40, 1.5–2.8 cm long, glabrous; outer filament whorl basally connate for 1–1.5 cm. Ovary 3-loculed, about 2.5–3.3 mm in diameter, tomentose. Styles 3, distinct, glabrous, 1.4–1.75 cm long. Capsule ovoid or subglobose, surface furfuraceous, 1.7–1.9 cm in diameter; pericarp ca. 2 mm thick.

##### Phenology.

Flowering in February, fruiting in June.

##### Etymology.

The specific epithet “shuangbaiensis” refers to Shuangbai County of Yunnan Province, the type locality of the new species.

##### Distribution and habitat.

Presently, *Camelliashuangbaiensis* is only known from its type locality, Shuangbai County of central Yunnan. It is distributed in mountain slope mixed forest habitat at altitudes of 1500–2000 m a.s.l.

##### Additional specimens examined.

China • Yunnan: Shuangbai County, Damaidi Town, Damaidi Country, 24°22.44'N, 101°50.84'E, 1740 m a.s.l., 5 February. 2024 (fl.), *S. Qu and G.P. Yang Lg2024133* (CNGB; SYS); • ibid., 11 June 2024 (young fr.), *S. Qu and F.F. Li Lg2024135* (SYS); • ibid., 11 June 2024 (young fr.), *S. Qu and F.F. Li Lg2024136* (SYS); • ibid., 11 June 2024 (young fr.), *S. Qu and F.F. Li Lg2024137* (SYS); • ibid., 11 June 2024 (young fr.), *S. Qu and F.F. Li Lg2024138* (SYS); • ibid., 11 June 2024 (young fr.), *S. Qu and F.F. Li Lg2024139* (SYS); • ibid., 11 June 2024 (young fr.), *S. Qu and F.F. Li Lg2024140* (SYS).

## ﻿Discussion

Morphologically, *Camelliashuangbaiensis* closely resembles *C.mileensis* and *C.hongkongensis*. However, it can be distinguished by its ovate leaves with tomentose adaxial surface, rounded leaf bases, and 14–16 bracteoles/sepals. Detailed morphological comparisons among *C.shuangbaiensis* and its relatives are presented in Table 1.

**Table 1.** Morphological comparison of *Camelliashuangbaiensis* and similar species.

Characters	*Camelliashuangbaiensis*	*C.mileensis*	*C.hongkongensis*
Habit	small trees, 2–4 m tall	shrubs to 2 m tall	trees to 10 m tall
Leaf blade	ovate to elliptic, abaxially tomentose, more or less punctate, pubescent along midvein, adaxially pubescent along midvein	elliptic, oblong, or lanceolate, abaxially sparsely villous along midvein, punctate, adaxially glabrous	oblong, oblong–elliptic, or oblong–lanceolate, both surfaces glabrous
Leaf length	3.5–6.5 cm	6–6.5 cm	6–12.5 cm
Leaf width	2.5–4 cm	2.5–3 cm	2–4 cm
Petioles	3–5 mm,pubescent	3–5 mm,hirtellous	7–13 mm, glabrous
Leaf margin	crenate–serrulate	serrulate	entire or obscurely undulate–denticulate
Leaf apex	acuminate to acute	bluntly and shortly caudate	acuminate to shortly acuminate
Leaf base	Rounded	cuneate to broadly cuneate	cuneate to obtuse
Bracteoles and sepals	14–16,semipersistent	9–10,semipersistent	11–12,semipersistent
Petals	6–7, red, obovate to broadly obovate, 1.2–1.9 × 1.4–3.5 cm	7–8, white or pale pink, obovate, 2–2.5 × 1.4–1.8 cm	6–7, red, broadly obovate, 3–3.5 × 1.5–2.3 cm
Stamens	filament whorl 1.5–2.8 cm,outer filament whorl basally connate for 1–1.5 cm	filament whorl 1.8 cm, outer filament whorl basally connate for 0.9–1.4 cm	filament whorl 2.5–3 cm,outer filament whorl basally connate for 1.3–2 cm
Styles	3, distinct, 1.3–1.7 cm	3,distinct, ca. 1.7 cm	3,distinct,2.8–3.3 cm
Ovaries	2.5–3 mm in diam., densely white tomentose	1.5 mm in diam., white tomentose,	ca. 2 mm in diam., densely tomentose
Fruit	1.7–2 cm in diam., furfuraceous, pericarp ca. 2 mm thick	1.2–1.5 cm in diam., furfuraceous, pericarp ca. 1.5 mm thick	2–3 cm in diam., furfuraceous, pericarp 3–4 mm thick

Section Furfuracea was initially circumscribed based on *C.furfuracea* (Merr.) Cohen-Stuart (Chang 1981), and primarily characterized by its furfuraceous (scaly) fruit surface. Ming (1999, 2000) merged this section into Sect. Heterogenea, however, molecular phylogenetic analyses (Xiao and Parks 2003; Vijayan et al. 2009; Zhao et al. 2023) have consistently rejected this taxonomic treatment, and a broader sect. Furfuracea including *C.hongkongensis* was supported.

Both *C.hongkongensis* and *C.shuangbaiensis* align with Sect. Furfuracea species in terms of their furfuraceous fruit surfaces and 3 free styles. However, their red petals clearly distinguish them from the species of Sect. Furfuracea.

Although *C.hongkongensis* was previously classified under Sect. Camellia (Chang 1981; Chang and Ren 1998; Ming et al. 2000; Ming and Bartholomew 2007), recent molecular studies (Xiao and Parks 2003; Vijayan et al. 2009; Zhao et al. 2023) have supported to place it within Sect. Furfuracea. Considering the morphological similarities between the two species, *C.shuangbaiensis* is likely also a member of Sect. Furfuracea.

*Camelliashuangbaiensis* is currently found only in the Damaidi Township, where it grows along the edges of dry-hot river valleys. With a population of just over 100 individuals, the species is restricted to areas adjacent to roadsides, which are heavily impacted by human activities. Additionally, some individuals have been observed to be parasitized by *Scurrula* species. Considering its restricted distribution and vulnerability to parasitic plants and invasive species, *C.shuangbaiensis* faces severe survival threats, which are further exacerbated by its proximity to vehicular pathways on mountain slopes.

These factors necessitate urgent and comprehensive conservation measures. Despite these challenges, the species’ striking floral characteristics render it a promising candidate for ornamental camellia breeding programs, highlighting the importance of implementing targeted ecological management strategies to ensure its long-term survival in situ.

## Supplementary Material

XML Treatment for Camellia shuangbaiensis

## ﻿Additional information

### Author ORCIDs

Zi-Yuan Li https://orcid.org/0009-0002-4941-0505

Bao-Huan Wu https://orcid.org/0000-0002-0498-0603

Shang Qu https://orcid.org/0009-0008-5531-5487

Le-Shan Du https://orcid.org/0000-0002-4418-4011

Hai-Ou Liu https://orcid.org/0000-0003-1148-1615

He-Xiang Duan https://orcid.org/0000-0003-1453-1403

Fei-Fei Li https://orcid.org/0000-0003-4733-6268

Wen-Hui Liu https://orcid.org/0009-0006-2043-6129

### Data availability

All of the data that support the findings of this study are available in the main text.
